# Concerted Evolution in the Ribosomal RNA Cistron

**DOI:** 10.1371/journal.pone.0059355

**Published:** 2013-03-12

**Authors:** Kershney Naidoo, Emma T. Steenkamp, Martin P. A. Coetzee, Michael J. Wingfield, Brenda D. Wingfield

**Affiliations:** 1 Department of Genetics, Forestry and Agricultural Biotechnology Institute, University of Pretoria, Faculty of Natural and Agricultural Sciences, Pretoria, South Africa; 2 Department of Microbiology and Plant Pathology, Forestry and Agricultural Biotechnology Institute, University of Pretoria, Faculty of Natural and Agricultural Sciences, Pretoria, South Africa; University of Ottawa, Canada

## Abstract

**Background:**

Gene conversion is the mechanism proposed to be responsible for the homogenization of multigene families such as the nuclear ribosomal gene clusters. This concerted evolutionary process prevents individual genes in gene clusters from accumulating mutations. The mechanism responsible for concerted evolution is not well understood but recombination during meiosis has been hypothesized to play a significant role in this homogenization. In this study we tested the hypothesis of unequal crossing over playing a significant role in gene conversion events within the ribosomal RNA cistron during meiosis, mitosis or both life stages in the fungal tree pathogen *Ceratocystis manginecans*.

**Methods:**

*Ceratocystis manginecans*, a haploid ascomycete, reproduces homothallically and was found to have two distinct sequences within the internally transcribed spacer (ITS) region of the ribosomal RNA cistron. The different ITS types were scored using PCR-RFLP assays and chi-square analyses to determine the level of significance of the changes in the ratios of the ITS types.

**Results:**

The relative ratios of the two ITS sequence types changed when the fungal isolates were cultured vegetatively or allowed to produced sexual structures and spores. These active changes were shown to occur more frequently during meiosis than mitosis.

**Conclusion:**

The evidence presented provides concrete support for homogenization in the rRNA gene clusters found in this fungus and that the most reasonable explanation for this process is unequal crossing over.

## Introduction

Current understanding of the structure and evolutionary history of genes encoding the RNA subunits comprising of ribosomes is based on research conducted over more than 40 years [1], [2], [3]. The small ribosomal subunit (18S) together with the large subunit RNAs (5.8S and 28S) are all processed from a single precursor RNA after it's transcription from the rRNA cistron. The ribosomal 5S gene is transcribed independently of the rRNA cistron and in many cases, is also located in the intergenic region of the rRNA cistron [4]. Within the cistron, the genes encoding the respective RNA subunits are separated by the internal transcribed spacer regions (ITS1 and ITS2), while the entire region is flanked by the 3′ and 5′ intergenic spacer regions [5]. Due to the high level of cellular demand for ribosomes, the rRNA cistrons occur in large head-to-tail tandem arrays at one or a few chromosomal loci [6]. However, through evolutionary time, the same DNA sequence is maintained in each cistron of these arrays, even though differences between the cistrons of different species are allowed to accumulate [7]. This is not consistent with classical evolutionary expectation, where all the members of a gene family would evolve independently [6]. Consequently, the term “concerted evolution” has been introduced to describe the form of evolution in which DNA or gene repeats evolve as single units in concert [1], [3], [6], [8], [9].

In their model of concerted evolution, Brown and colleagues [10] proposed that the homogenization process in DNA or gene repeats requires the individual repeats to evolve in a manner which is dependent on one another [6]. In this way the transmission and accumulation of mutations occurring in the repeat region becomes homogenized (i.e., mutations spread throughout the rRNA repeat array to all the member cistrons). Although the exact mechanisms determining concerted evolution remains unclear [3], two fundamental processes are thought to drive the homogenization process; unequal crossing over and gene conversion [11], [12]. The essential difference between these two evolutionary forces is that gene conversion maintains the copy number of a gene at a constant size, while unequal crossing over has the potential to cause fluctuations in the gene copy number from one generation to the next [13], [14]. Unequal crossing over represents a form of homologous recombination between repeats or cistrons, located at dissimilar positions within a locus on two chromosomes or between different cistrons on the same chromosome [1], [15]. Gene conversion results in unidirectional or non-reciprocal DNA transfer between DNA duplexes [2] (at the same or different loci) due to homologous recombination that was initiated by DNA double stranded breaks [16], [17]. The process of unequal crossing over can arise during mitosis within or between sister chromatids or during meiosis within or between homologous or non-homologous chromosome pairs [1]. The effects of gene conversion have also been observed during both meiosis and mitosis [1], [16].

Most evidence for concerted evolution of the rRNA cistron is derived from research using metazoan model organisms (e.g., *Drosophila* and *Xenopus*) and microorganisms such as *Saccharomyces cerevisiae* [1], [6], [10]. Evidence for this process can also be inferred from DNA sequence data available from a variety of plants and animals [18], [19]. The growing number of genomes accessible for study has provided additional support for the existence of concerted evolution [2]. Empirical evidence for the involvement of unequal crossing over in the homogenization of rRNA cistrons is also available from a range of eukaryotes, particularly *S. cerevisiae* [1]. However, experimental evidence for the contribution of gene conversion in the concerted evolution of DNA or gene repeats is mostly restricted to the homogenization of protein-encoding gene families [16], [20].

Although various studies have considered concerted evolution in non-model plants [21] and animals [22], few studies have focused on the homogenization of multicopy genes and rRNA cistrons in non-model fungi [22], [23]. For fungi, the rRNA cistron is of primary importance because various regions of the rRNA cistron are frequently targeted for DNA-based identification [2]. For example, the ITS region is utilized as the standard DNA barcoding region for fungal identification [24]. The potentially far-reaching consequences of concerted evolution on fungal taxonomy and diagnostics (i.e., by influencing the sequence and evolutionary trajectories as well as the resulting phylogenies of the rRNA cistron), requires a more detailed understanding of this process in fungi.

**Figure 1:**
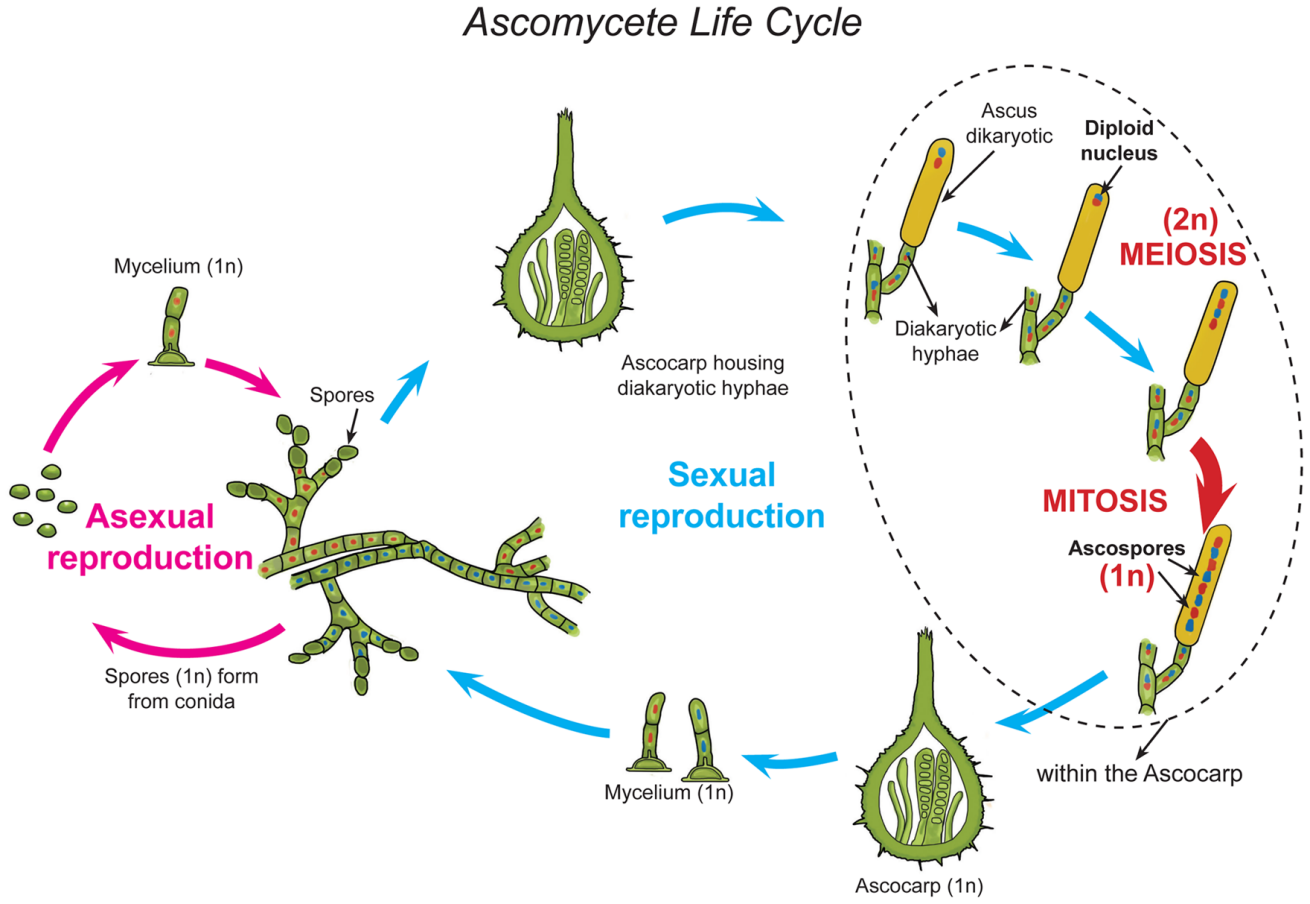
Life cycle of a *Ceratocystis* species. Shown here for this typical ascomycete includes both sexual (meiotic) and asexual (mitotic) cycles. In this case both the meiotic and mitotic states occur in a single haploid culture, a condition known as homothallism in fungi.

*Ceratocystis manginecans* is a homothallic ascomycete and an important plant pathogen [25]. In the laboratory environment, this fungus is maintained in culture by repeatedly transferring mycelial strands from agar cultures. Its homothallic nature results in the production of sexual structures without the requirement of outcrossing [25] (**Figure 1**). Therefore, in a single isolate, both sexual and asexual reproduction occurs, without a change to the genetic structure of the organism during either mitosis or meiosis.

In recent studies, some isolates of *C. manginecans* were identified that contain two distinct rRNA ITS variants, a phenomenon that has also been observed in other fungi [26], [27] as well as some higher eukaryotes [2]. In addition, we have seen some variation in the ratios of these ITS variants while maintaining these fungi in culture. These observations and the reproductive strategy of *C. manginecans* in the laboratory provided an opportunity to investigate concerted evolutionary processes in this fungus. Our specific aims were to determine whether homogenization or a drift-like variation of the rRNA cistron occurred during meiosis, mitosis or both life stages and whether the effects of this process could be seen as fluctuations of the different ITS types. We were able to provide statistical evidence of significant changes occurring within the rRNA cistron during both meiosis as well as mitosis. These changes could be linked to concerted evolution and ultimately to the occurrence of unequal recombinational crossing and potentially gene conversion of rRNA cistrons.

## Materials and Methods

### Fungal Isolates

Two sets of four *C. manginecans* isolates were used in this study. The first set of isolates (CMW 13581, 13584, 23641 and 23643) apparently harbour a single ITS type (**Figure S1**), while those in the second set harbour two types of ITS (CMW 13852, CMW 17568, CMW 17570 and CMW 23635) [25]. All isolates were maintained on 2% malt extract agar (MEA: 20% w/v; Biolab, Midrand, South Africa) at 25°C.

**Figure 2:**
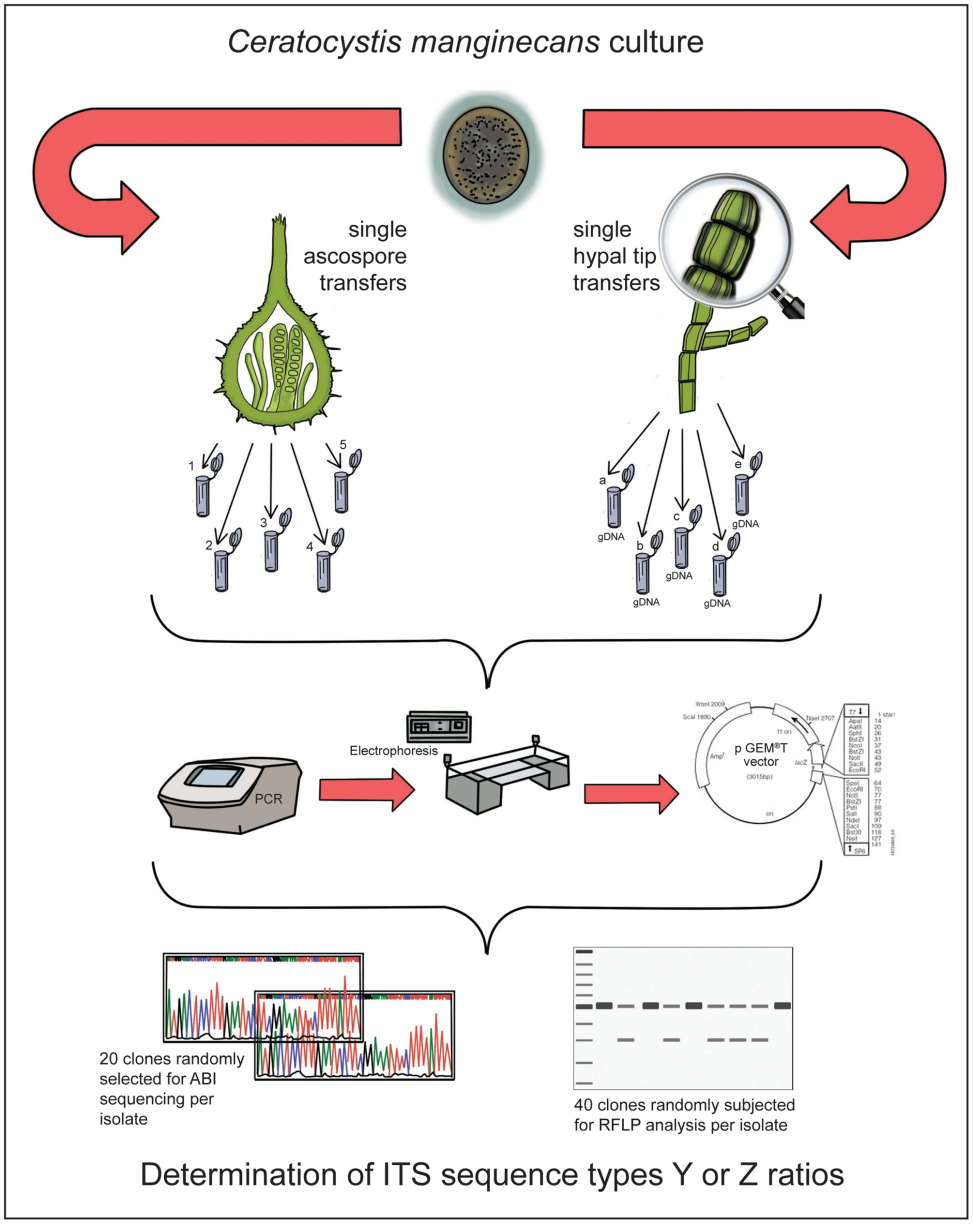
Diagrammatic representation of the methodology employed in the experimental design. The fungus *Ceratocystis manginecans*, isolate CMW 17568 was derived from single meiospores (ascospores) to generate the meiotic progeny, and the four sequential single hyphal tip isolations generated the mitotic generations of the fungus.

The second isolate set was used to test whether homogenization of rRNA cistrons occurred during mitosis. For this purpose five sequential sub-cultures for each of the four isolates were prepared, which was done by transferring a single hyphal tip of each isolate to fresh MEA medium and allowing it to grow for approximately two weeks. The entire process was repeated an additional four times, each time using hyphal tips from the new sub-culture (**Figure 2**). All of these isolate sets, together with the original isolates, have been deposited in and can be obtained from the culture collection (CMW) of the Tree Protection Cooperative Programme, Forestry and Agricultural Biotechnology Institute, University of Pretoria, Pretoria, South Africa.

The sexual spores produced by each of the four isolates in the set harbouring two ITS types were used to test whether homogenization of the rRNA cistrons occurred during meiosis. This was accomplished by allowing the four isolates to produce sexual structures (perithecia) by incubating them on MEA at 25°C for one week. From the mature sexual structures (perithecia) that developed, a single ascospore (meiospore) mass was collected for each isolate after which individual ascospores were used to provide the DNA template for the amplification of the ITS region (**Figure 2**).

### ITS PCR and sequencing

DNA was isolated from all cultures and subcultures and used as templates in PCRs with primers ITS1 and ITS4 [5] using protocols described previously [28], [29]. DNA was not isolated from ascospores but the ITS region was amplified directly from single ascospores using PCR. To control for the possible impact of PCR artifacts, a number of thermostable DNA polymerases were tested. These included Expand *Taq* [Roche Diagnostics, Mannheim, Germany], FastStart *Taq* [Roche Diagnostics, Mannheim, Germany], and SuperTherm *Taq* [Fermentas, Inqaba Biotechnical Industries (PTY) LTD., South Africa].

All amplicons were cloned using the pGEM-T Vector system II [Promega, Anatech Instruments, South Africa]. In each case, cloned inserts were amplified directly from 20 randomly selected recombinants using vector-specific primers and PCR as described previously [30]. After purification with Sephadex® G-50 columns (SIGMA), the products were sequenced in both directions using the original vector-specific primers, the Big Dye terminator version 3.1 cycle sequencing kit (Applied Biosystems, Foster City, CA) and an ABI PRISM ™ 3100 Autosequencer (Applied BioSystems, Foster City, California, USA). All sequences were then manually aligned using BioEdit [31] to visualize the two ITS types or variants [referred to as Y and Z] (**Figure 2**).

### ITS PCR-RFLPs

In order to analyse the large number of cloned inserts that were generated, a PCR-RFLP (Restriction Fragment Length Polymorphism) technique was developed to differentiate between the two ITS sequence types. The ITS region of the one sequence type [type Y, GenBank accession number KC261853], contained the restriction site recognised by the enzyme *Tsc*AI [Fermentas, Inqaba Biotechnical Industries (PTY) LTD., South Africa] in two positions. This site is present only once in the other ITS sequence type [type Z, GenBank accession number KC261852]. The restriction enzyme, *Tsc*AI was thus used to differentiate between the two ITS sequence types. Briefly, the PCR-RFLP technique employed involved direct amplification of 40 randomly selected recombinants using vector-specific primers and PCR as described above. The PCR products were then digested with *Tsc*AI according to the supplier's specifications. The RFLP fragments were stained with Gel Red [Anatech Instruments, South Africa], separated using agarose (3%, w/v, Supplier) gel electrophoresis and visualized under UV trans-illumination.

### Statistical Analysis

Chi-square analyses [28] were conducted to establish whether the ratios of the different ITS sequence types were statistically different in the sub-cultures that had been produced, as well as in the single ascospores that were subjected to PCR. A 99.999% level of confidence was applied. The number of degrees of freedom (df  = 4) was established based on the number of transfers made (i.e., n = 5, thus n−1 = 4). The null hypothesis was that no significant change would be observed between the ratios after meiosis or mitosis.

The combined chi-square statistic tested the same null hypothesis under the conditions of mitosis and meiosis. Each sub-cultured isolate thus served as a replica test allowing for all the individual chi-square analyses to be combined. The probability values for the combined statistics were calculated using the initial chi-square value gained from the original replica tests for the respective isolate and then correlating this to the associated lowest possible probability in the R-statistical programming software [32]. This software allows for the lowest mathematical probability to be determined as an inverse of the chi-square derivative. The logarithm of this probability is then cumulatively added for all the replicas to generate a total probability value, which can then be checked against the number of degrees of freedom. In this case, the number of degrees of freedom is established by taking twice the number of separate tests and probabilities carried out initially (thus 2*k*  = 2(4)  = 8 degrees of freedom). A 99.999% level of confidence was also applied.

## Results

### Scoring of the ITS types

Use of the universal ITS primers [5] yielded PCR products of 646 base pairs (bp) in length. Sequence analysis of *C. manginecans* isolates CMW 13581, 13584, 23641 and 23643 confirmed that they all harboured a single ITS type (**Figure S1**), while isolates CMW 13582, 17568, 17570 and 23635 contained a combination of both ITS types. After cloning the latter ITS fragments into the pGEM®-T Easy vector, and sequencing the insert, no differences could be ascribed to the polymerase used for amplification. In all the various sequence sets (i.e., sequences generated for the 20 cloned inserts of the five vegetative culture sets for each of the four original *C. manginecans* isolates, as well as the five single-ascospores from the four isolates) examined, we observed two different ITS types (Y and Z) (**Figure S2**).

PCR-RFLP analysis of the 40 randomly selected *Escherichia coli* clones generated from a particular isolate and its sub-cultured replicates or single ascospores showed the expected RFLP profiles. The size of the undigested PCR fragment from the *E. coli* clone was 920 bp. Digestion of the PCR clone products of ITS sequence type Z yielded a doublet of 460 bp. The ITS sequence type Y produced three fragments [525 bp, 335 bp and 60 pb], although the smallest fragment was too small for regular detection using agarose gel electrophoresis.

### Statistical analysis of the ITS types

For each of the isolates, CMW 13582, 17570, 17568 and 23635, a set of 600 cloned ITS fragments were analysed (i.e., 60 for each of five vegetative cultures and 60 representing the amplicons generated from five single ascospores). The observed Z:Y ratios for the ITS types in each set of 60 cloned amplicons after meiosis and mitosis are summarized in supplementary information (**Table S1**). To determine the chi-square statistic, the ITS type frequency for each original isolate (i.e., the expected frequency) was compared with the observed frequency after meiosis and mitosis (**Table S1**). While it is possible that there could be more than two ITS types, these would need to exist at a frequency of less than one percent, not to have been observed in our analyses. It was anticipated that the copy number of the rRNA cistron is around 120 to 130 per cell [33], which makes the possibility of missing additional variants negligible.

In each isolate, there was a significant oscillation in the relative frequencies of the different ITS variants (**Table S1**). For example, the ascospore-derived data for CMW 17570.1 showed 36 ITS Z types and 24 ITS Y types, while those for CMW 17570.2 had 7 ITS Z and 53 ITS Y. These dramatic changes were observed less often in the mitotically-derived data. For example, in isolates CMW 17570.a and CMW 23635.a, the proportion of the ITS type Z variant remained lower than the ITS type Y variant (**Table S1)**. At a 99.999% level of confidence, the chi-square analysis showed that the frequency of the two ITS types changed significantly during both meiosis and mitosis. These analyses also showed that the ITS type ratios changed more often as a consequence of meiosis (**Table 1**).

**Table 1:** Summary of Chi-Square values across the replica tests for the respective isolates^a^.

Replica Isolate	Meiosis χ^2^value^b^	Mitosis χ^2^value^c^
χ^2^_0.01 [4*]_ = 13.28^d^		
CMW 13582	**18.34**	**19.32**
CMW 17568	**24.92**	11.99
CMW 17570	**26.11**	3.04
CMW 23635	**24.11**	2.21

a For each isolate the corresponding degrees of freedom(df)* was calculated as (n−1), where n is the number of transfers per isolate.

b, c Combined replica data for respective meiosis and mitosis chi-square values as determined in supplementary information (**Table S1**). These data are based on five sequential rounds of either mitotic transfers or sexual crosses for each isolate.

d The corresponding chi-square value when a 99.999% level of confidence is applied at 4 degrees of freedom. The level of significance was established on the basis of the χ^2^prediction been greater or less than the respective replica isolate χ^2^value. Thus, values in bold denote significant levels of change, whilst the clear blocks denote non-significant change.

Contrast to what was observed for isolates CMW 17570, 17568 and 23635, the results for isolate CMW 13582 showed a significant change in frequency of the ITS types in both the meiotic and mitotic sets of observations (**Table 1**). For each of the meiotic and mitotic sets of observations, a combined chi-square value was also obtained [28] (**Table 2** **,** **Table 3**). This value considered each isolate as a separate replicate of the experiment and probability values were obtained using R programming software [32]. This approach employed the prediction of probability statistics by making use of the initial chi-square values. Overall, the values obtained for the meiotic observations for the different isolates produced a highly significant deviation at a 99.999% level of confidence, indicating that these ITS type frequencies were not due to a chance sampling event.

**Table 2:** Combined Chi-Square value statistics across all replicas for meiosis^a^.

Replica Isolate	Meiosis χ^2^ value^b^	Probability Value^c^	ln (P)^d^_;_ [28]
**CMW 13582**	18.34	0.010	−4.605
**CMW 17568**	24.92	5.221×10^−5^	−9.860
**CMW 17570**	26.11	3.007×10^−5^	−10.412
**CMW 23635**	24.11	7.592×10^−5^	−9.486
•At 2*k*, where *k* = the number of separate tests and probabilities, thus degrees of freedom = 2(4) = 8			−2 ∑ ln(P) = 68.726*
χ^2^_0.001 _ [_8_] = **68.726>1.214×10^−12^, thus highly significant** [32]			

a The different fungal isolate serves as a replica of the same statistical test for meiosis.

b Chi-square value as determined from the original replica isolates as shown in supplementary information (**Table S1**) for that specific isolate.

c Lowest possible probability score associated to that chi-square value determined by R-statistical software algorithmic programming [32].

d The logarithmic value of the probability**^c^**. ^*^The formula used to derive the sum of all the separate tests for each replica.

• The number of degrees of freedom is established by taking twice the number of separate tests and probabilities carried out initially (thus 2*k*  = 2(4)  = 8 degrees of freedom). A 99.999% level of confidence was applied.

**Table 3:** Combined Chi-Square value statistics across all replicas for mitosis^a^.

Replica Isolate	Mitosis χ^2^ value^b^	Probability Value^c^	ln (P)^d^_;_ [28]
**CMW 13582**	19.32	0.0007	−7.264
**CMW 17568**	11.98	0.0175	−4.046
**CMW 17570**	3.04	0.551	−0.596
**CMW 23635**	2.21	0.7155	−0.335
•At 2*k* where *k* = the number of separate tests and probabilities, thus degrees of freedom = 2(4) = 8			**−2 ∑ ln(P) = 24.482** *
χ^2^_0.001 _ [_8_] = **24.482 >0.00186, thus highly significant** [32]			

a The different fungal isolate serves as a replica of the same statistical test for mitosis.

b Chi-square value as determined from the original replica isolates as shown in supplementary information (**Table S1**) for that specific isolate.

c Lowest possible probability score associated to that chi-square value determined by R programming [32].

d The logarithmic value of the probability**^c^**.

* The formula used to derive the sum of all the separate tests for each replica.

• The number of degrees of freedom is established by taking twice the number of separate tests and probabilities carried out initially (thus 2*k*  = 2(4)  = 8 degrees of freedom). A 99.999% level of confidence was applied.

## Discussion

Concerted evolution has been hypothesized to be the result of gene conversion or unequal cross over during recombination. In this study, we have provided direct evidence of unequal recombination and the potential of gene conversion occurring within the rRNA cistron of the fungal pathogen *C. manginecans*. The observed changeability in the ratios of the Z:Y ITS types is a direct consequence of unequal cross over during recombination, while the lack of a second ITS type likely emerged because a non-reciprocal recombination between cistrons [1]. Our results showed that the processes of concerted evolution are highly dynamic as reflected by the dramatic changes in the ratios of the two ITS types analysed. This is similar to what was observed by Ganley and Kobayashi [15] who used a tagged *S. cerevisiae* rDNA unit to monitor the frequency of duplication and deletion events. In *C. manginecans*, however, we were able to show the dynamic flux of different ITS types in both mitosis and meiosis against an identical genetic background without the need to tag the ITS region. Concerted evolutionary processes could, therefore, be directly linked to unequal crossing over events occurring during both the meiotic and mitotic lifecycles within the rRNA cistron of *C. manginecans*.

The fluctuations observed in the frequencies at which both the ITS Z and Y variants occurred across the four *C. manginecans* isolates examined, suggest an associated fluctuation in the overall copy number of the rRNA cistron in these isolates. Similar evidence of fluctuations in the rRNA copy number has been recorded in other eukaryotes such as *Drosophila melanogaster* [4], [14], [34], [35]. However, the need for large amounts of rRNA to be produced in a cell probably precludes the copy numbers of these genes ever becoming inordinately low [13]. Ide et al. [33] had demonstrated in their study of *S. cerevisiae* that an increase or decrease in the copy number of a highly transcribed gene led to increased sensitivity to DNA damage and increased cell toxicity, respectively. Various authors have suggested that a specific mechanism acts to maintain the multicopy nature of highly transcribed genes [36]. This previous work on *S. cerevisiae* thus suggests that a similar multicopy maintenance mechanism probably functions to preserve the 120 to 130 copies (authors unpublished) of the rRNA cistron in *C. manginecans*.

In this study, the effects of concerted evolution were more pronounced after meiosis than mitosis. Statistical analyses of the frequencies of the two ITS types examined, indicated that the ratios changed more often and sometimes more dramatically as a consequence of meiosis than mitosis. In CMW 13582, for example, the ratio of the ITS types Z and Y changed from 55∶5 in one generation to 9∶51 in the subsequent meiotic generation. This change in ratio was found in a single ascospore and was thus the result of a single meiotic event. In order to generate an ascospore having a 9∶51 ratio of the two ITS variants, a reduction of the rRNA cistron copy number to around 12 copies would be required, if this were the result of a single cell division. Ascospore development in fungi such as *C. manginecans* involves three cell divisions, the first two are those normally observed during meiosis and then there is an additional cell division to produce eight ascospores. But, whichever way the problem is examined, at least 4 divisions would be needed to change from a ratio of 55∶5 to 9∶51 (**Figure S3**), if the copy number of the cistron remained the same or was in some way constrained (**Figure S3A**). However, if there is no restriction placed on the cistron copy number then the reduction in the ITS sequence types can occur in just one division (**Figure S3B**). This, therefore, suggests that significant reduction in the copy number of the cistrons can occur as a consequence of unequal recombination.

Although the occurrence of multiple ITS types in *C. manginecans* suggests, at first glance, a relaxation in the mechanisms driving concerted evolution of the rRNA cistron, there are other more plausible explanations for their occurrence. For example, in their seminal work on the rRNA cistrons of humans and other primates, Arnheim et al. [34] showed that some regions of this unit remain polymorphic and that these polymorphisms are maintained through evolutionary time by natural selection. But unlike in the primate situation, all individuals of *C. manginecans* do not harbour both types of cistron (**Figure S1**). This is probably because the *C. manginecans* polymorphisms examined in this study do not represent alleles that were conserved during evolution. An alternative hypothesis would be that ITS types Y and Z represent polymorphisms that were united into the same genome following an interspecies hybridization event. In fact, intraspecific polymorphisms in the rRNA cistron are commonly thought to be a property of hybrid species [27], [37], [38]. Following this hypothesis, the dynamic processes of concerted evolution led to the loss of the second ITS type from some isolates of this fungus (CMW 13581, 13584, 23641 and 23643).

Unequal crossing over, though considered to be rare during mitosis, has previously been reported to occur during this life stage [39]. It was demonstrated that *S. cerevisiae* tagged mutants underwent mitotic recombination during the interphase process of the cycle replication [40]. These sister chromatids were found to be recombining and resulted in the production of diploid cells [41]. The mitotic chromosomes thus allow for the formation of homolog pairs, which greatly enhance the ease of recombination due to the formation of synaptonemal complexes [42]. Literature also verifies that crossing over events are driven by various genes acting in unity to achieve the variation [42]. Studies have, however, not been able to show empirical evidence for the unequal crossing over event actively occurring during the cellular divisions of meiosis and mitosis within the same biological organism [42]. The results of this study, therefore, provide evidence that recombination occurs in a non-reciprocal manner and that the overall concerted evolution of the rRNA cistron is mediated in a random and not directed fashion.

**Figure 3:**
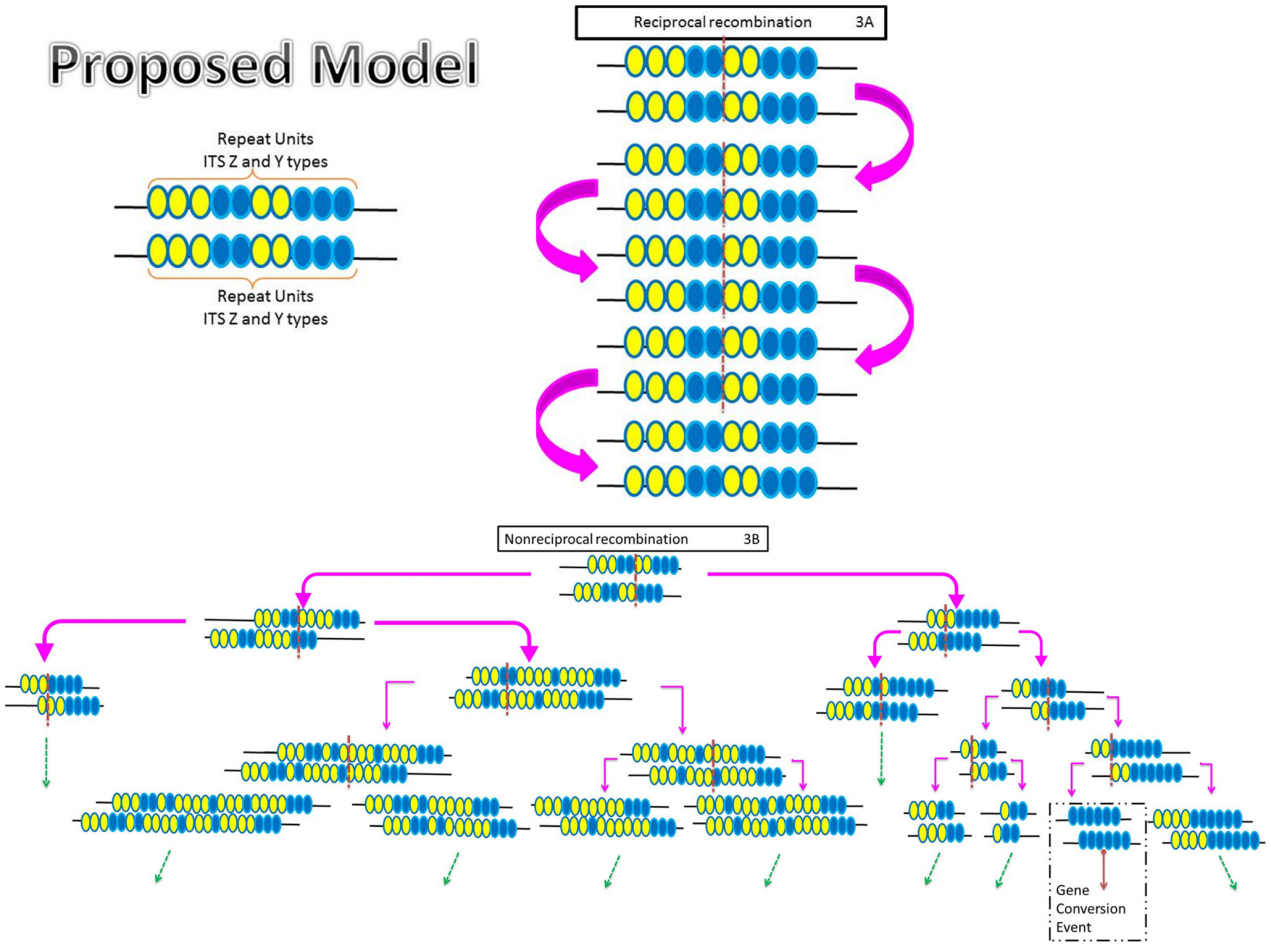
Diagram illustrating a proposed model of concerted evolution in *Ceratocystis manginecans*. Concerted evolution is seen as a result of both gene conversion and unequal crossing over occurring during both the meiotic and mitotic life cycles of *Ceratocystis manginecans* on a hypothetical chromosome which contain both ITS type Z and Y sequences. Yellow circles represent ITS type Z sequence repeat units whilst blue circles represent ITS type Y sequence repeat units. Crossing over (broken red lines) is shown hypothetically as reciprocal recombination (3A) or nonreciprocal recombination (3B) events, in each case the repeat units are subjected to gain and loss events which can result in Gene conversion (solid red line). The broken green arrows indicate multiple unequal crossing events.

A model is proposed to explain our results using unequal crossing over during recombination, (**Figure 3**) in a hypothetical situation where there are 20 copies of the same repeat unit of the haploid fungus *C. manginecans* that reproduces homothallically. If the repeat units recombine only in a reciprocal process (**Figure 3A**) then the ratios of the different ITS types would have remained static. In order to explain the differing ratios observed in this study, it is necessary to propose that non-reciprocal crossing over occurs (**Figure 3B**). It is thus possible that the copy number of these multigene families can be dramatically reduced or expanded. However, there would be selection against any individual cell in which the copy number drops below a minimum level to sustain cell function, while there is selection against cells containing more than a certain maximum number of copies. Via this dynamic process, the copy number of this multicopy element is thus maintained at some ideal number and the gene sequences continuously homogenised to maintain identical (or near identical) sequences in each unit. Ultimately, these unequal crossing over events have the potential to result in gene conversion (**Figure 3**, Gene conversion Event) and thus loss of one or the other ITS type.

Previous models addressing concerted evolutionary processes have commonly been based on hybrid organisms containing multiple copies of the ITS repeat units which homogenize over the generations through meiotic and mitotic cellular divisions [37]. The present study also considered an organism in which isolates had two different ITS sequences. The fact that this fungus exists in a haploid state, a situation common to many fungi but unique to most other eukaryotes, and the fact that it is able to undergo sexual reproduction without outcrossing (homothallic) provided us with a unique opportunity to observe experimentally changes in the ratios of the different rRNA types during meiosis and mitosis. Other than the ITS ratios for isolates CMW 17570, 17568 and 23635, which did not change significantly in their mitotic lifecycles, the results suggest that the repeat units of the rRNA cistron can undergo significant size changes during relatively few cell divisions as was the case for isolate CMW 13582. Thus our presented model summarises the overall observations and illustrates how concerted evolution is a consequence of unequal recombination which ultimately over time leads to gene conversion.

## Supporting Information

Figure S1 **Aligned DNA sequence.** Screen print showing the aligned DNA sequences of only ITS sequence type Y in *Ceratocystis manginecans* isolates CMW 13581, 13584, 23641 and 23643.
(TIF)

Figure S2 **Aligned DNA sequence.** Screen print of aligned DNA sequences showing the differences in the sequences of the two ITS sequence types (Z and Y) in *Ceratocystis manginecans* CMW 17568.
(TIF)

Figure S3 **Hypothetical rRNA cistron showing meiotic and mitotic divisions.** Illustration of a hypothetical situation for *Ceratocystis manginecans* where we assume that its rRNA cistron has 600 copies undergoing meiotic and mitotic divisions. In this example, under meiotic conditions each division represents either an increase or a decrease in the ITS sequence types. Figure S3A depicts a restricted cistron size scenario whilst Figure S3B has no size restriction.
(TIF)

Table S1 **Summary of the observed ITS sequence types from 60 cloned amplicons across all test replicas for both meiosis and mitosis based on five sequential rounds of either mitotic transfers or sexual crosses for each isolate◂.**
(TIF)
