# Supplementary figures and images for: Concerted Evolution in the Ribosomal RNA Cistron
Source: PLoS One. 2013 Mar 12;8(3):e59355. doi: 10.1371/journal.pone.0059355 (PMC3595265; doi:10.1371/journal.pone.0059355)

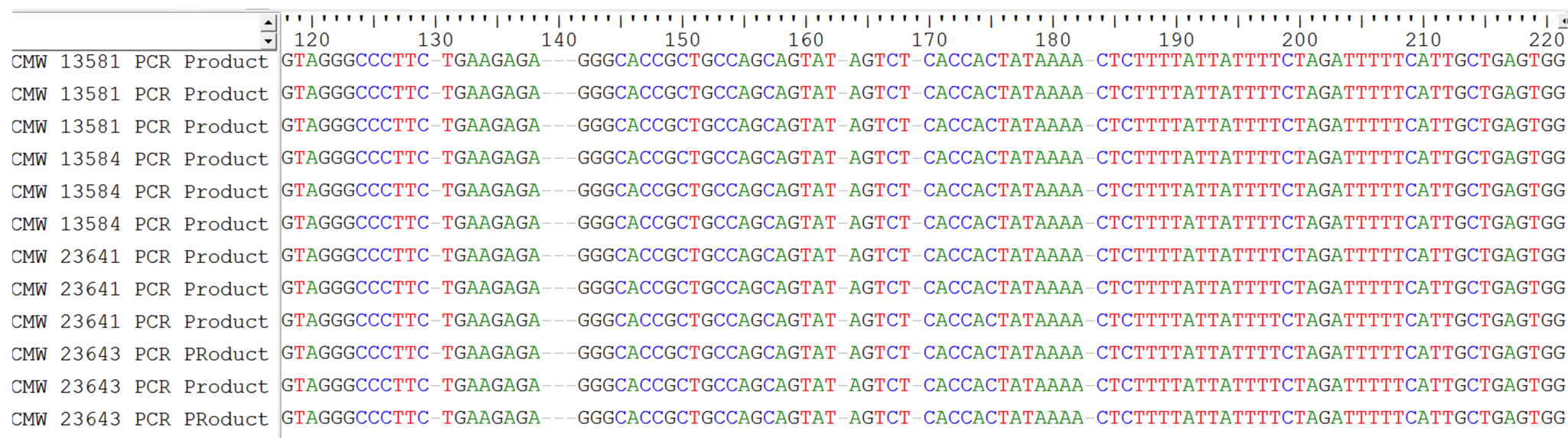

Supplement: Figure S1 — Aligned DNA sequence. Screen print showing the aligned DNA sequences of only ITS sequence type Y in Ceratocystis manginecans isolates CMW 13581, 13584, 23641 and 23643. (TIF) [file pone.0059355.s001.tif]

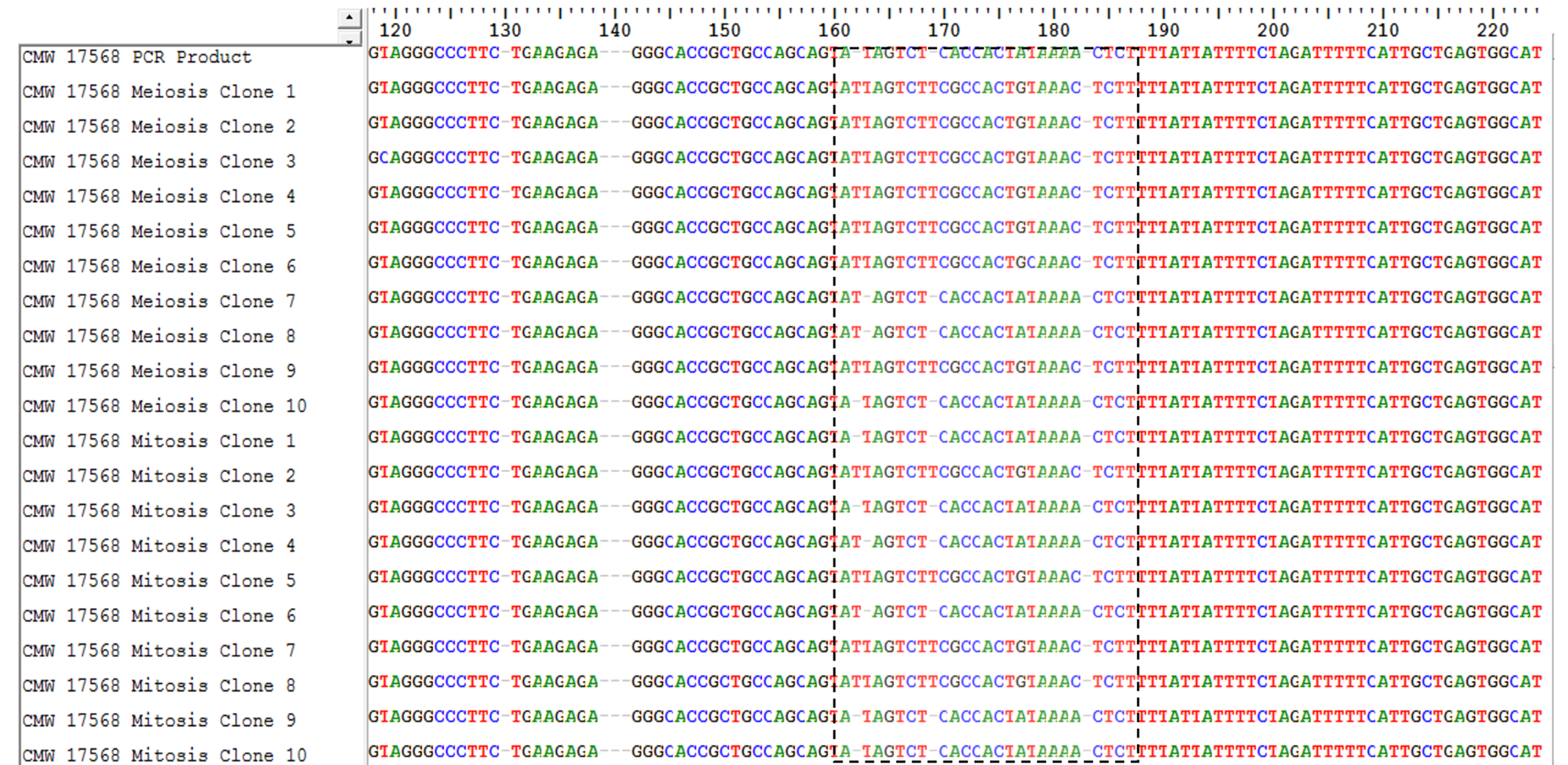

Supplement: Figure S2 — Aligned DNA sequence. Screen print of aligned DNA sequences showing the differences in the sequences of the two ITS sequence types (Z and Y) in Ceratocystis manginecans CMW 17568. (TIF) [file pone.0059355.s002.tif]

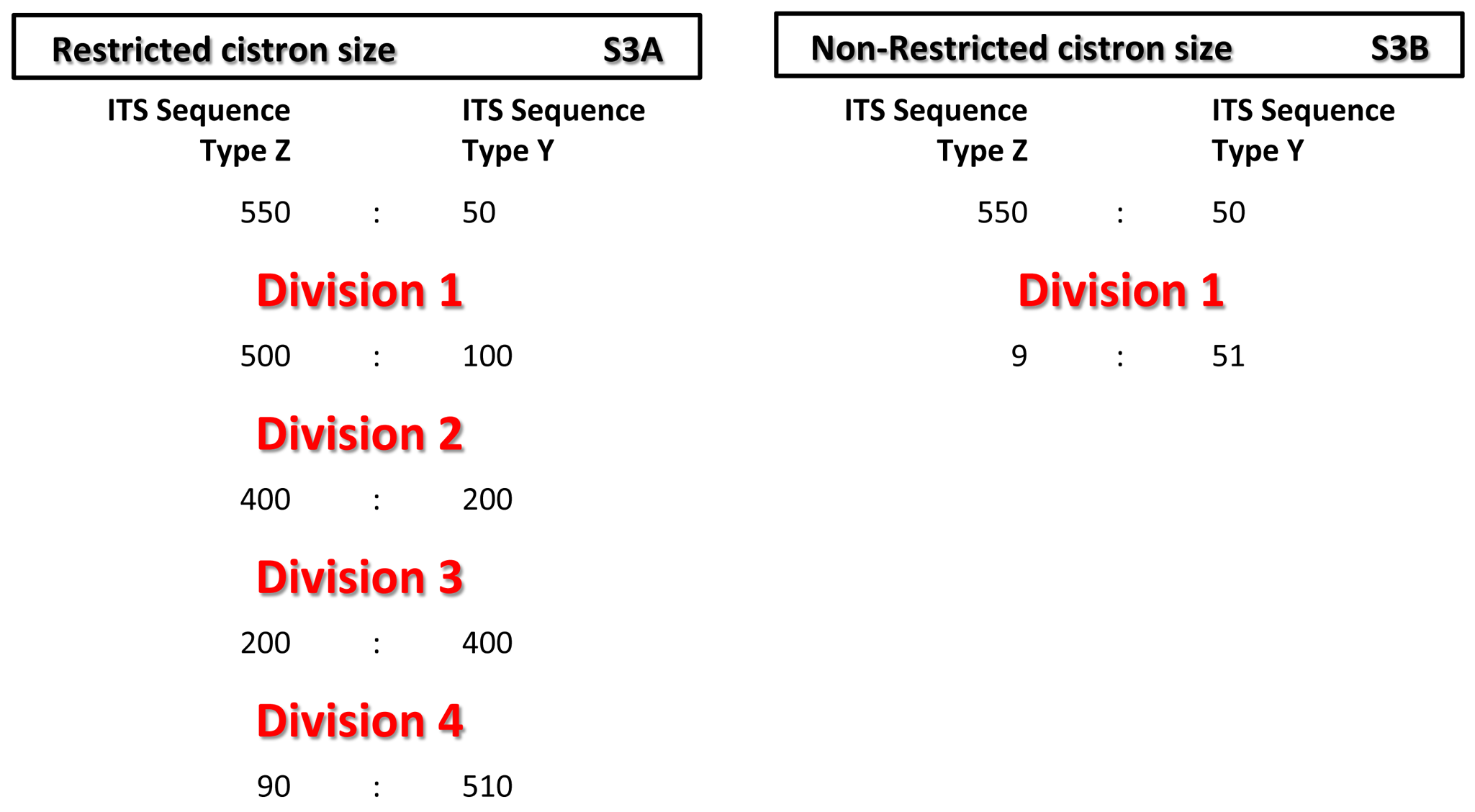

Supplement: Figure S3 — Hypothetical rRNA cistron showing meiotic and mitotic divisions. Illustration of a hypothetical situation for Ceratocystis manginecans where we assume that its rRNA cistron has 600 copies undergoing meiotic and mitotic divisions. In this example, under meiotic conditions each division represents either an increase or a decrease in the ITS sequence types. Figure S3A depicts a restricted cistron size scenario whilst Figure S3B has no size restriction. (TIF) [file pone.0059355.s003.tif]

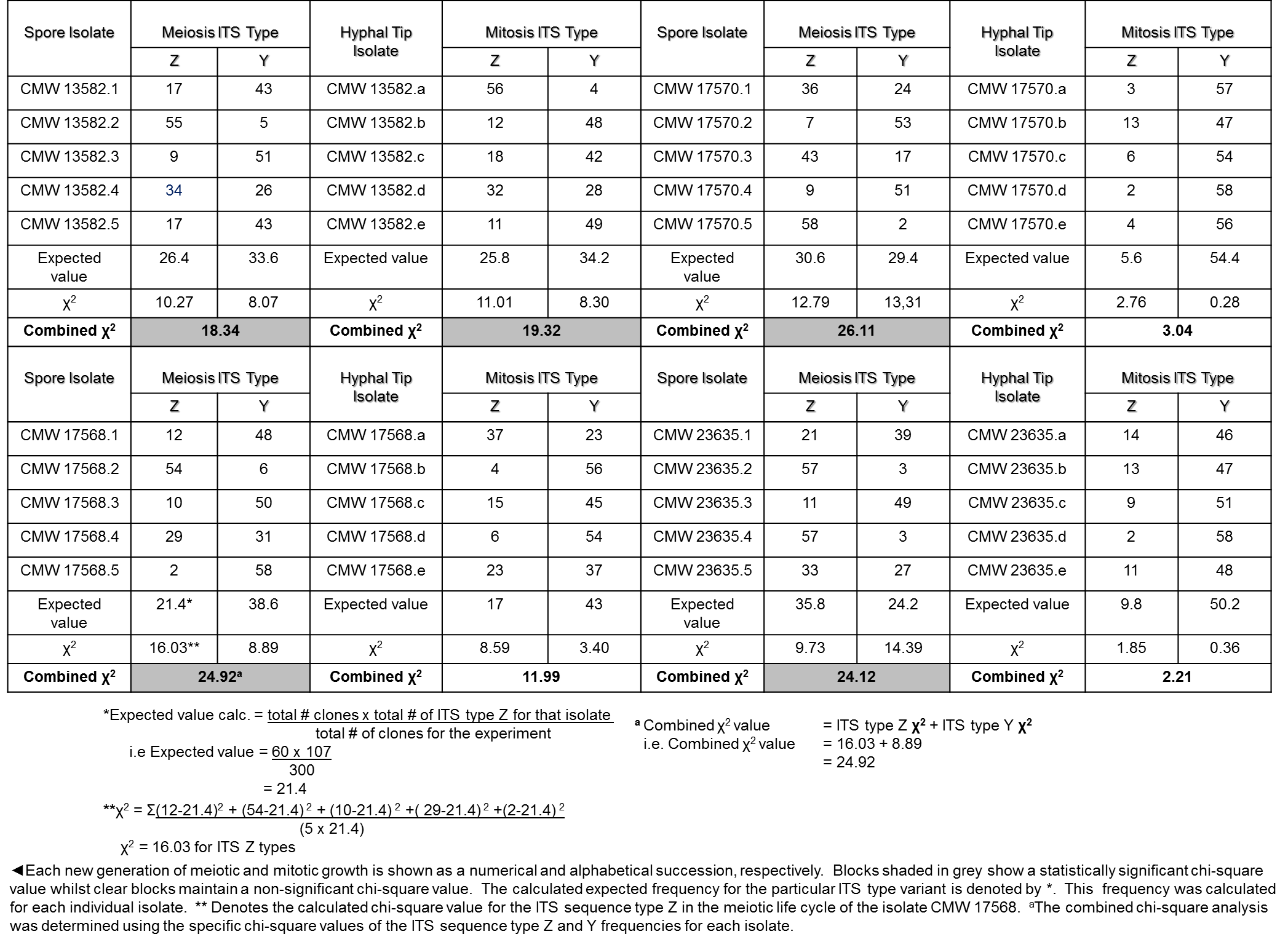

Supplement: Table S1 — Summary of the observed ITS sequence types from 60 cloned amplicons across all test replicas for both meiosis and mitosis based on five sequential rounds of either mitotic transfers or sexual crosses for each isolate◂. (TIF) [file pone.0059355.s004.tif]
